# Topography and malaria transmission heterogeneity in western Kenya highlands: prospects for focal vector control

**DOI:** 10.1186/1475-2875-5-107

**Published:** 2006-11-10

**Authors:** Andrew K Githeko, John M Ayisi, Peter K Odada, Francis K Atieli, Bryson A Ndenga, John I Githure, Guiyun Yan

**Affiliations:** 1 Climate and Human Health Research Unit, Centre for Vector Biology and Control Research, Kenya Medical Research Institute, P. O. Box 1578 Kisumu 40100, Kenya; 2 Human Health Division, International Centre for Insect Physiology and Ecology, P. O. Box 30772, Nairobi, Kenya; 3 Programme in Public Health, College of Health Sciences, University of California, Irvine, CA 92697, USA

## Abstract

**Background:**

Recent resurgence of malaria in the highlands of Western Kenya has called for a more comprehensive understanding of the previously neglected complex highland vector ecology. Besides other drivers of malaria epidemiology, topography is likely to have a major effect on spatial vector and parasite distribution. The aim of this study was to determine the effects of topography on malaria spatial vector distribution and parasite prevalence.

**Methodology:**

Indoor resting adult malaria vectors and blood parasites were collected in three villages along a 4 km transect originating from the valley bottom and ending at the hilltop for 13 months. Members of the *Anopheles gambiae *complex were identified by PCR. Blood parasites were collected from children 6–13 years old and densities categorized by site of home location and age of the children.

**Results:**

Ninety eight percent (98%) of *An. gambiae s.s*. and (99%) *Anopheles funestus *were collected in houses located at the edge of the valley bottom, whereas 1% of *An. gambiae s.s*. were collected at mid hill and at the hilltop respectively. No *An. funestus *were collected at the hilltop. Malaria prevalence was 68% at the valley bottom, 40.2% at mid hill and 26.7% at the hilltop. Children aged six years and living at the edge of the valley bottom had an annual geometric mean number of 66.1 trophozoites for every 200 white blood cells, while those living at mid-hill had a mean of 84.8, and those living at hilltop had 199.5 trophozoites.

**Conclusion:**

Malaria transmission in this area is mainly confined to the valley bottom. Effective vector control could be targeted at the foci. However, the few vectors observed at mid-hill maintained a relatively high prevalence rate. The higher variability in blood parasite densities and their low correlation with age in children living at the hilltop suggests a lower stability of transmission than at the mid-hill and valley bottom.

## Background

The prevalence of malaria in the highlands of Eastern Africa varies spatially and temporally as a result of seasonal weather changes, climate variability [1,2] and topography [3]. The topography of the highlands comprises hills, valleys and plateaus. Rivers and streams run along the valley bottoms in the valley ecosystem and swamps are a common feature. Unlike in lowland plains, where drainage is poor and mosquito breeding habitats have an extensive distribution, the majority of breeding habitats in the hilly highlands are confined to the valley bottoms because the hillside gradients provide efficient drainage [4]. The non-homogeneous distribution of larval breeding habitats is likely to affect adult vector spatial distribution and may, consequently, lead to focal malaria transmission and heterogeneous human exposure to malaria. It can thus be expected that the malaria immunity profile in the highlands is influenced not only by age [5], but also by distance from the foci of transmission. The pattern of malaria transmission in the highland plateau ecosystems may be less distinct due to the flat topography and the more diffuse hydrology resulting from numerous streams. Heterogeneity in transmission can lead to highly variable stability of malaria transmission in space with some areas having stable and others unstable transmission. This would lead to different sensitivities to epidemics within relatively short distances in the highlands.

Malaria epidemics in the Western Kenya highlands have been recorded since the early 1940's [6,7] and currently the malaria situation is getting worse partly due to resistance to anti-malarial drugs and lack of vector control measures [8,9]. Furthermore, it has been demonstrated that malaria epidemics in the Western Kenya highlands are partly driven by climate variability [10-13]. The impact of malaria epidemics on human morbidity and mortality may become more severe because climate variability is predicted to become more frequent and intense [14]. Development of efficient and cost-effective methods for epidemic prevention, reduction of morbidity and mortality is urgently needed.

The heterogeneity of transmission has important implications for vector and morbidity control. Understanding the spatial pattern of vector distribution provides opportunities for limited and thus more cost-effective control programs [15]. For example, due to the large areas affected by epidemic malaria, it is not possible to spray every house with indoor residual insecticides. Knowledge of transmission foci would also lead to a better understanding of spatial distribution of the stability of transmission and risk of severe disease. This would enable a more rational application of interventions in areas of varying malaria exposure risk. Recent theoretical consideration of heterogeneous vector distribution and targeted control have arrived at similar conclusions [16-18].

Many vector and parasite studies assume a random distribution of mosquitoes and malaria cases. While this may be largely true in the lowlands such an assumption is not true in the highlands. An assumption of random distribution would lead to erroneous sampling. Where distribution of events is contiguous in space and time it would be advisable to use stratified sampling procedures.

This study was undertaken to determine the effects of topography on distributions of vectors and malaria infections in Kakamega district, Western Kenya.

## Methods

### Study site

**Figure 1:**
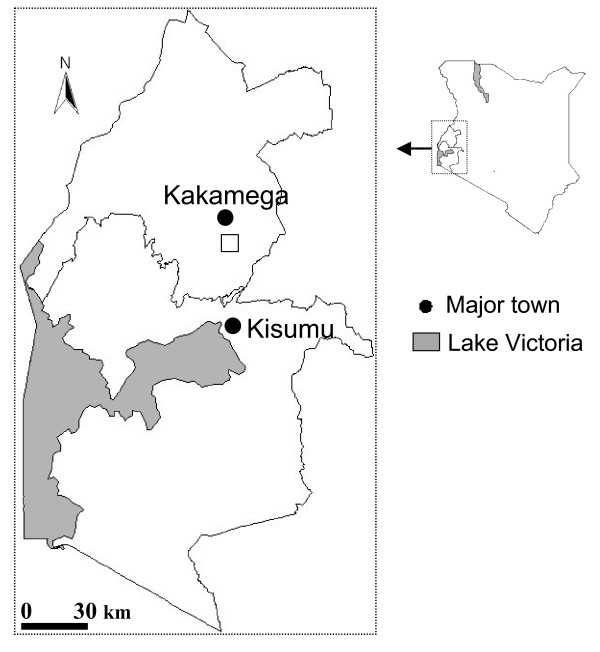
A map showing the study area in Western Kenya.

The study was carried out at Iguhu Location, 0° 17'N, 34° 74'E, at an elevation of 1,450–1,580 m above sea level in Kakamega district in Western Kenya (Figure 1) from February 1999 to February 2000. The mean annual rainfall is 2,000 mm and the mean annual daily temperature is 20.8°C.

### Field sampling

**Figure 2:**
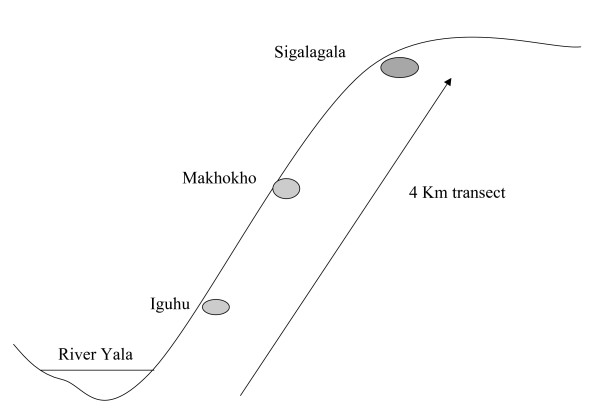
Location of the three villages along the transect.

A short transect was established, originating from the bottom of the Yala River valley and terminating at Sigalaga village, 4 km uphill (Figure 2). Nine houses were randomly selected: three houses near Yala River (Iguhu village), three mid-hill (Makhokho village), and three houses, 4 km at hilltop (Sigalagala village). Mosquitoes were collected from these houses three times a month for thirteen months using the pyrethrum spray collection method (PSC). The mosquitoes were transported to the Kenya Medical Research Institute laboratories in Kisumu, and morphologically identified as *Anopheles gambiae sensu lato (s.l.) *or *Anopheles funestus*, the only two vectors collected indoors in the area. Their gonotrophic status was also identified and recorded. A proportion of the *An. gambiae s.l*. females were analyzed by the polymerase chain reaction (PCR) method to determine the proportion of the sibling species of *An. gambiae s.s*. and *Anopheles arabiensis *in the sample [19].

### Estimation of entomological inoculation rates (EIR)

The EIR was estimated from the fraction of freshly blood fed females collected in the houses [20]. Houses used for the vector collections were two-roomed and occupied by bachelors, thus only one person slept in the house most of the times. Sporozoite rates used for the estimation, were obtained for the period beginning in 2003 to the end of 2004 [21] from mosquitoes collected from the same villages as in this study.

### Parasite prevalence and density

Two cohorts of 120 children aged 6–13 years old living in the three villages (Iguhu, Mahkohko, and Sigalagala) and attending Iguhu and Makhokho primary schools were identified for monthly malaria parasite prevalence surveys during the study period. After explanation of the study's objectives, methodologies, risks, and benefits, informed consent to take part in the study was obtained from the children's parents. The study was approved by the Ethics Review Committee of the Kenya Medical Research Institute (KEMRI), Protocol KEMRI SSC# 469. The two cohorts were alternated for monthly infection prevalence surveys to avoid sampling fatigue. Sampling was performed using the finger-prick method to obtain thick and thin smears. Immediately after the finger prick, any child presenting with a fever, had his or her thick-blood smear stained in 10% Giemsa for 10 min in the field and examined for the presence of malaria parasites. If parasites were seen, the child was treated with an analgesic (Paracetamol) and an appropriate dose of pyrimethamine/sulfadoxine combination (Fansidar^®^). Each child was asked in which of the three villages his or her home was located. The parasite data were classified according to the location of the child's home.

All other slides were stained in 4% Giemsa for 30 min in the laboratory. Parasites were identified to species by morphology and infection intensity was determined by counting the number of infected red blood cells against 200 leucocytes.

### Data analysis

Data were entered onto a Quattro Pro V8 spreadsheet and thereafter analyzed with reference to the three villages along the transect. The proportion of vectors collected and children infected with malaria in each village was determined. The geometric mean annual indoor resting vector density and the 95% confidence interval for *An. gambiaes.s*. collected at the valley bottom were calculated.

Age-specific blood parasite density of *Plasmodium falciparum *for each village was log-transformed to stabilize variance. Regression equations for parasite density versus age of children were determined for each village. Mean monthly parasite densities for children populations living in the three villages (pooled samples) were calculated and tested for differences among the village means by a single factor ANOVA. Because not all children were regularly tested for malaria the data were not suitable for repeated sampling ANOVA. Detailed examination of the parasite prevalence data indicated the presence of parasites particularly in children residing in the valley bottom were not independent events. This violates assumptions in most statistical tests. Data reported only refers to malaria infections as indicated by presence and number of parasites in blood. As other causes of fever were not assessed we do not include any clinical malaria cases in the analysis. Monthly variations in parasite densities for the different villages were indicated by the 95% confidence intervals.

## Results

### Vector spatial distribution

Out of the 90 samples of *An. gambiae s.l*. analysed by PCR all were found to be *An. gambiae s.s*. The majority of *An. gambiae s.s*. vectors (98.2%) were collected at the valley bottom while 0.8% and 1.0% were collected in houses at mid-hill and hilltop, respectively (Table 1). The geometric annual mean number of *An. gambiae s.s*. collected in houses at the valley bottom was 7.4 females per house and the 95% confidence interval was 2.4–12.0. However, in May 1999 a mean of 59.6 females/per house were collected in houses at the valley bottom. A monthly average of less than or equal to one vector was collected at mid hill and at the hilltop. In terms of proportions, a similar pattern was observed in *An. funestus*. *An. gambiae s.s*. comprised 87.8% and *An. funestus *12.2% of vectors collected in the three villages.

**Table 1:** Proportions of *An. gambiae s.s*. and *An. funestus *adult females collected in houses along the transect

Vector species	Valley bottom	Mid-hill	Hilltop	Total number collected and %
Elevation (M)	1450	1500	1580	
*An. gambiae s.s*.	98.1	0.83	1.07	1681 (87.9%)
*An. funestus*	99.1	0.9	0.0	234 (12.1%)

### Entomological inoculation rates

Later studies on vectors collected between 2003–2004 in the same study site reported mean annual sporozoite rates of 3.6% for *An. gambiae s.s*. and 5.2% for *An. funestus *[21]. The EIR for *An. gambiae s.s*. collected at the valley bottom was 10.6, mid-hill 0.0 and hilltop 0.04 infectious bites/person/year. The EIR for *An. funestus *was 2.2 at the valley bottom, 0.05 at mid-hill and 0.00 at hilltop ib/p/y. The total estimated EIR at the three locations was 12.8, 0.05 and 0.04 ib/p/y at valley bottom, mid-hill and hilltop respectively.

### Parasite prevalence and density

Malaria prevalence is hyper-endemic at the valley bottom, meso-endemic at mid-hill, and close to hypo-endemic at the hilltop (Table 2). This endemicity profile translates to stable transmission at the valley bottom and unstable transmission at the hilltop while the mid-hill village was in an intermediate state. A linear relationship was found between *P. falciparum *prevalence in the study group and altitude (R^2 ^= 0.98). There was a 15.9% reduction in prevalence for every 50 meter increase in altitude along the transect.

**Table 2:** Proportion of *Plasmodium *trophozoite species and *P. falciparum *gametocytes along the transect

	Proportion of infected children with parasites (%)		
Parasite	Valley bottom (1450 m)	Mid-hill (1500 m)	Hilltop(1580 m)

*P. falciparum*	68.0	40.2	26.7
*P. falciparum gametocytes*	5.8	5.9	3.0
*P. malariae*	1.6	0.8	0.0
*P. ovale*	3.0	2.3	1.7

Parasite prevalence of *P. malariae *and *P. ovale *were less than 4% and do not seem to play a significant role of malaria in the study sites (Table 2)

**Figure 3:**
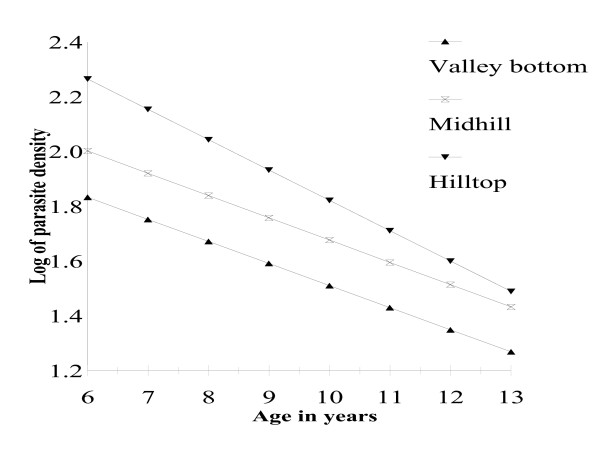
Trends in log transformed *Plasmodium falciparum *densities in blood with age and altitudinal location on the transect.

Parasite densities declined inversely with the age of the children irrespective of the distance or elevation of the home from the valley bottom, as indicated by the significant negative correlation between parasite densities and age of the children (Figure 3). There was also a village-specific rate of parasite density decline, which is an indication of differential exposure to malaria transmission. For example, while children aged six years (Figure 3) and living at the valley bottom had a geometric mean annual number of 66.1 trophozoites for every 200 white blood cells, those living at mid-hill had a mean of 84.8, and those living at hilltop had 199.5 trophozoites.

**Figure 4:**
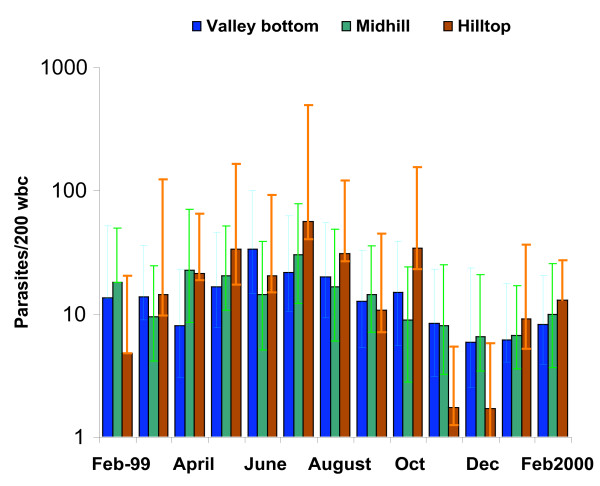
Temporal variation in blood parasite density with 95% confidence intervals along the transect (Y-axis in log scale).

Children living at the bottom of the valley and at mid-hill were able to control the numbers of parasites in the blood during a period of increased exposure to infected mosquitoes in the main transmission season. In contrast, children living at the top of the hill developed two-fold more parasites in July during the main transmission season compared to the children living at the lower sites. A similar event was observed during the short transmission season in October 1999 (Figure 4). These children had a greater variability in the blood parasite density as indicated by the magnitude of the error bars (Table 3 and Figure 4) and the correlation between age and parasite density was lowest in this group (Table 3).

**Table 3:** Regression statistics of *Plasmodium falciparum *blood densities along the transect

Site	Regression statistics			
	Intercept	Age (X) co-efficient	R	Standard error of Y estimate

Valley bottom	2.3	-0.08	0.73	0.2
Mid hill	2.5	-0.08	0.77	0.18
Hilltop	2.9	-0.11	0.48	0.53

At the valley bottom, children aged nine years old had the equivalent parasite density as children aged 13 years living at hilltop as shown graphically in Figure 3. However, analysis of variance on the mean parasite densities in children living at the valley bottom, mid-hill and hilltop indicated that the three human populations were not significantly different in their ability to control the numbers of parasites in the blood (p = 0.39) as age increased. Log transformation of the parasite density may mask real statistical differences and consequently the absence of a statistically significant difference should be taken with caution.

## Discussion

The topographic features of the highlands restrict the spatial distribution of vector breeding habitats. Later work in the same site has demonstrated that larval breeding habitats are confined to the valley bottom [22-24] and hence a low intensity of exposure to malaria in hilltop residents resulting in a non-homogeneous parasite burden and probably the incidence of morbidity. This unique epidemiology has important implications in the development of malaria control strategies. Furthermore, it indicates that failure to take topographic effects on transmission into consideration can lead to biased data on the spatial distribution of malaria prevalence in the highlands.

The presence of a high proportion of *An. gambiae s.s*. in this region has resulted from creation of breeding habitats due to reclamation of natural swamps for agriculture use [22]. Early in the 1970s it was observed that *An. funestus *was as an important vector of malaria in the highlands [7]. Clearing swamps and creating drainage channels for agriculture may negatively affect the abundance of *An. funestus *and at the same time favor the abundance of *An. gambiae s.s*. Due to *An. gambiae*'s shorter generation time in aquatic habitats, larger populations of this species can be expected compared to those of *An. funestus*. As a result, a relatively larger vector population exists, followed by a higher risk of malaria transmission.

The EIR at mid-hill was 257-fold and that at the hilltop 320-fold lower than that of the valley bottom. This difference in magnitude is not reflected in the parasite prevalence or the difference in parasite density. Studies in North Eastern Tanzania came to similar conclusions [25].

The valley bottom in the study area was characterized by well-established transmission, with the majority of children 6–13 years old maintaining asymptomatic parasitemia. Data indicate that under these conditions the children were able to suppress high parasitemias and presumably avoid severe disease. However, severe disease in hyper-endemic areas is more frequent in children under three years old [5]. Age-matched children living under much less transmission pressure at the hilltop had a higher parasitemia and were possibly more susceptible to severe disease, which includes anemia and cerebral malaria. The most significant effect of distance from the foci of transmission was the delay in the ability to suppress parasite density. Children at higher elevation took longer to achieve the same level of parasite density as children at lower elevation. The precision of the spatial distribution of parasite densities could have been improved by the use of the global positioning system and geographic information system technologies. Further studies on the relationships between land use changes in the area to malaria transmission using these technologies are currently underway.

While the population living in and near the valley maintains a large reservoir of infectious gametocytes, the people living away from this area comprise a high proportion of susceptible individuals. Under permissive climatic conditions the infectious vector population could increase, leading to higher rates of malaria prevalence. Susceptible individuals are at a high risk of infections that could lead to severe disease, particularly when many of the anti-malarial drugs are losing their efficacy.

Despite the very low numbers of vectors collected at mid-hill and a subsequently low EIR, a large number of children (40%) were infected. This indicates a high efficiency of transmission by the vectors. Although the possibility that children at mid-hill acquired their infections at the valley bottom cannot be ruled out, this seems unlikely because children generally sleep at their homes. It is acknowledge, however, that vector sampling did not represent all the areas where the children were living. The implication of these results is that any vector control method will have to be highly effective because even very low vector densities can maintain a high level of prevalence.

While vector control is necessary for malaria control, data from this study suggest that reduced transmission could result in the human population having less ability to suppress blood parasite density. Such a shift in the immune status could increase the risk of severe disease and greater mortality. It is critical that vector control programmes such as long-term use of insecticide impregnated bed nets be sustainable so that the risk of severe disease is reduced [26].

An alternative approach to sustainable vector control is focused intermittent indoor residual spraying specifically designed to reduce the impacts of potential epidemics. This approach would require an efficient epidemic forecasting tool, which would identify the potential epidemic with sufficient lead time to allow time for indoor residual insecticide spraying [10].

The El Niño climate variability phenomenon is characterized by positive temperature anomalies and above normal rainfall in the Western Kenya highlands – conditions associated with increased malaria transmission and indeed epidemics [10]. The warming phase of the El Niño is normally identified several months before malaria transmission occurs, and this would allow vector control operations to be planned and executed before the beginning of a hyper-transmission event. Results from this study indicate that transmission in the valley ecosystem is associated with focal points close to the valley bottoms.

Data from this study have shed light on how limited indoor residual spraying could be effectively applied to control focally transmitted malaria. Assuming that the flight range of *An. gambiaes.s*. and that of *An. funestus *are 2 km and 1 km respectively [27] and that 98% of the vectors are confined to this boundary, then the indoor residual spraying need only be applied to this area. Such a targeted approach would considerably reduce the cost of the intervention. Early vector control campaigns in the Nandi (1955–1957) and Kericho (1945–1949) districts in the highlands of western Kenya with DDT and dieldrin, respectively, virtually eradicated malaria [7].

## Authors' contributions

AKG, JMA were involved in the study design, data collection analysis and manuscript writing. PKO FKA, JIG were involved in the data collection, entry and manuscript writing. BAN carried out PCR analysis of the vectors. GY carried out a critical analysis of the data and the manuscript.
